# A novel technique to assess rotational deformities in lower extremities using CT-based motion analysis

**DOI:** 10.1038/s41598-021-00532-y

**Published:** 2021-10-26

**Authors:** Peyman Bakhshayesh, Ugwunna Ihediwa, Sukha Sandher, Alexandros Vris, Nima Heidari, Anders Enocson

**Affiliations:** 1 Karolinska Institutet Karolinska University Hospital, Stockholm, Sweden; 2 Royal London Hospital, Barts Healthcare NHS Trust, London, UK

**Keywords:** Experimental models of disease, Translational research

## Abstract

Rotational deformities following intramedullary (IM) nailing of tibia has a reported incidence of as high as 20%. Common techniques to measure deformities following IM nailing of tibia are either based on clinical assessment, plain X-rays or Computed Tomography (CT) comparing the treated leg with the uninjured contralateral side. All these techniques are based on examiners manual calculation inherently subject to bias. Following our previous rigorous motion analysis and symmetry studies on hemi pelvises, femurs and orthopaedic implants, we aimed to introduce a novel fully digital technique to measure rotational deformities in the lower legs. Following formal institutional approval from the Imperial College, CT images of 10 pairs of human lower legs were retrieved. Images were anonymized and uploaded to a research server. Three dimensional CT images of the lower legs were bilaterally reconstructed. CT-based motion analysis (CTMA) was used and the mirrored images of the left side were merged with the right side proximally as stationary and distally as moving objects. Discrepancies in translation and rotation were automatically calculated. Our study population had a mean age of 54 ± 20 years. There were six males and four females. We observed a greater variation in translation (mm) of Centre of Mass (COM) in sagittal plane (95% CI − 2.959–.292) which was also presented as rotational difference alongside the antero-posterior direction or Y axis (95% CI .370–1.035). In other word the right lower legs in our study were more likely to be in varus compared to the left side. However, there were no statistically significant differences in coronal or axial planes. Using our proposed fully digital technique we found that lower legs of the human adults were symmetrical in axial and coronal plane. We found sagittal plane differences which need further addressing in future using bigger sample size. Our novel recommended technique is fully digital and commercially available. This new technique can be useful in clinical practice addressing rotational deformities following orthopaedic surgical intervention. This new technique can substitute the previously introduced techniques.

## Introduction

The main aim of lower limb orthopaedic surgery is restoration of normal anatomy and function. Rotational deformities following IM nailing of tibia has a reported incidence of 20%^1^. Common techniques to measure deformities following IM nailing of tibia are either clinical examination, plain X-ray or Computed Tomography (CT) with manual calculation comparing the treated leg with the uninjured contra lateral side^2–4^. All these proposed techniques are dependent on manual calculation with risk for inter- and intra-observational discrepancies and bias^5^.

Further pre-operative planning is widely employed in elective and emergency orthopaedic surgery for its numerous benefits, including optimization of implant placement and simplification of intraoperative decision making. Trauma complicates the planning process by altering anatomy, obscuring the blueprint from which direct inferences can be made^6–8^.

The inherent symmetry of skeletal features represents a potential solution to the problem of templating in trauma. The femur has been shown to be usefully symmetrical and reliable as a template for contralateral injury, although some controversy exists^6,9,10^.

Other significant similarities between the lower limbs relevant to preoperative templating have been observed. For instance, the hip-knee angle (HKA), lateral distal femoral angle (LDFA), medial proximal tibial angle (MPTA), posterior proximal tibial angle (PPTA) and posterior distal femoral angle (PDFA) have been reported to be symmetrical features within the same individual^11^.

Some authors have found right-to-left intra-subject variations in characteristics such as the shape of the tibial tuberosity and tibial and fibular shaft diameters^9,12^.

Moreover, various imaging methods have been used in the literature, generating the potential for non-uniform estimation of symmetry or otherwise^5^. Most of these current techniques are using X-ray or CT-scan with further manual calculation subject to lack of reproducibility of reference points with inherent inter- and intra-observer measurement reliability issues^6^.

CT is preferred over planar imaging techniques, such as plain radiographs, in evaluation of shape symmetry due to its acquisition of three-dimensional detail^13^.

There are previous rigorous researches using 3D images of the bone or implants and merging of the surface anatomies to report either excessive motion over time or mismatch in anatomy^6–8,14–17^.

The primary aim of this study was to introduce a novel practical technique, CT-based motion analysis (CTMA), to measure lower leg rotational deformities based on previous rigorous research. The second aim of this study was to see whether lower legs of healthy human adults are symmetrical using our proposed technique with previously proven high precision and accuracy^6,8^.

## Material and methods

This study was modelled on a previous similar works on femur and pelvis analysis^6–8^. Study protocol was approved by the Imperial College Healthcare and NHS Medical Research Council prior to conduction. All methods were performed in accordance with the relevant guidelines and regulations of Imperial College Healthcare and NHS England. Written consent was retrieved from all individuals pertinent to trusts policy. We used Picture Archiving and Communication System (PACS) at St Mary´s Hospital, London, UK. In patients with no lower limb injuries, we randomly selected ten consecutive lower extremity CT images captured between January and December 2018.

A 256-slice Philips Brilliance CT scanner (Koninklijke Philips N.V., Amsterdam, The Netherlands) using contrast-enhanced CT scans was used. The CT scanner´s gantry was AirGlide. The CT scan acquisition parameters were: the aperture 700 mm, the focus-isocenter distance 570 mm, the Focus-detector distance 1040 mm, the rotation time 0.27 s with collimation of 2 × 128 × 0.625 mm, the Field of View 200–500 mm and the matrix 512. The contrast medium used in this cohort was 70-mL of Omnipaque (General Electric Healthcare, Chicago, IL, USA) which was administered intravenously. The Package Filter was iDose4 Premium. In average the tube voltage was 100 kV and the tube current 89–134 mAs. Average radiation dose was 520–920 mGy cm. All images were downloaded as DICOM files. All files were anonymized using Sectra© package (Sectra, Linköping, Sweden). These were coded and kept safe. Images were transferred to a research server. Images were reconstructed in 3D using a 3D Trauma package (Sectra, Linköping, Sweden) creating STL files. Segmentation of the right and left lower legs were performed. Mirroring of the left lower leg was performed using the CTMA package offered by Sectra^6^.

We uploaded images from the server, downloading them to the research data, creating 3D STL files and fusion process for each case took roughly 3 days.

**Figure 1:**
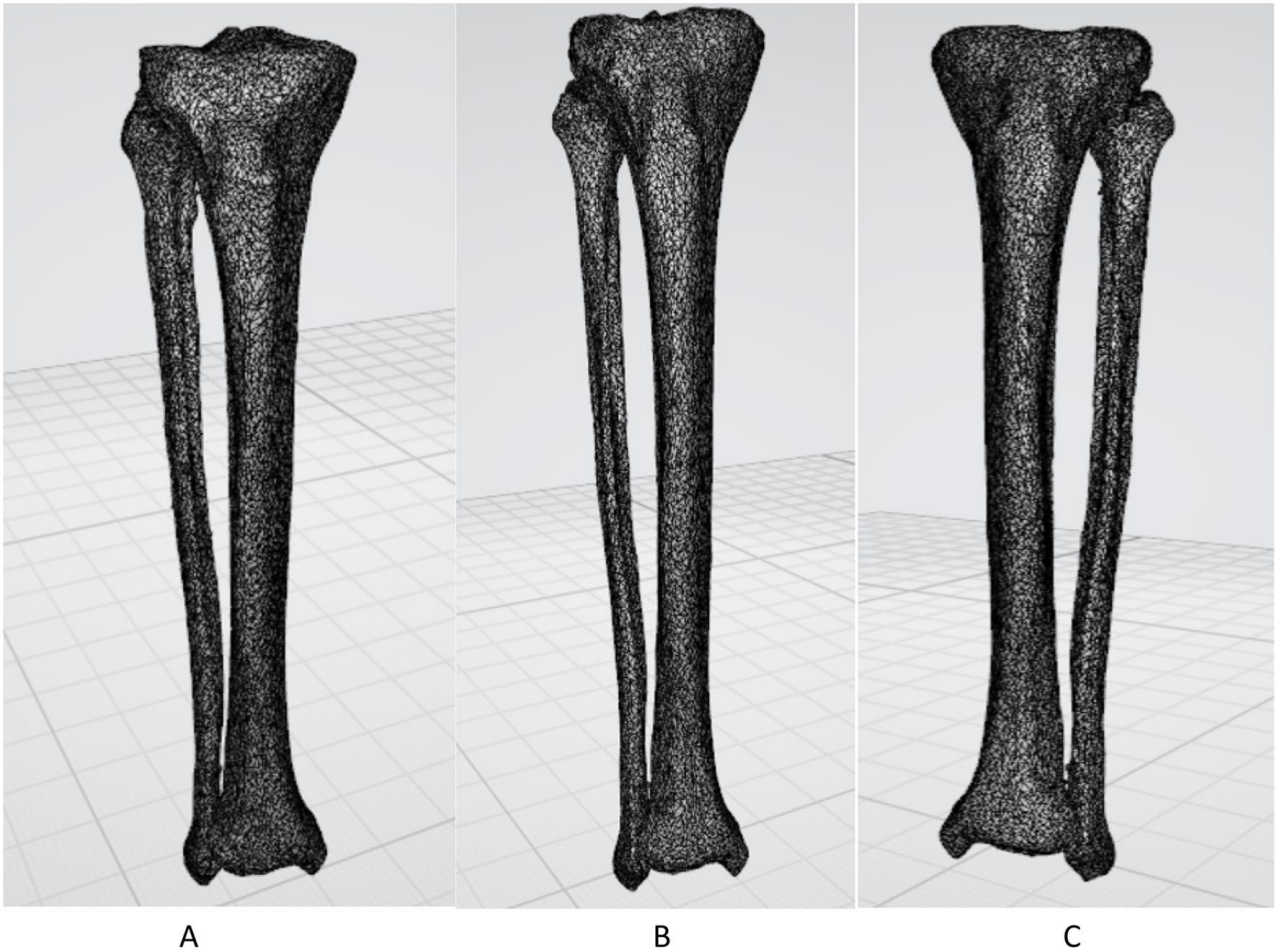
(**A**) Illustrates 3D image of right lower leg. (**B**) Illustrates the mirrored image if the left lower leg. (**C**) Illustrates the left lower leg.

Images of the right lower limb and mirrored images of left lower limb were saved (Fig. 1).

**Figure 2:**
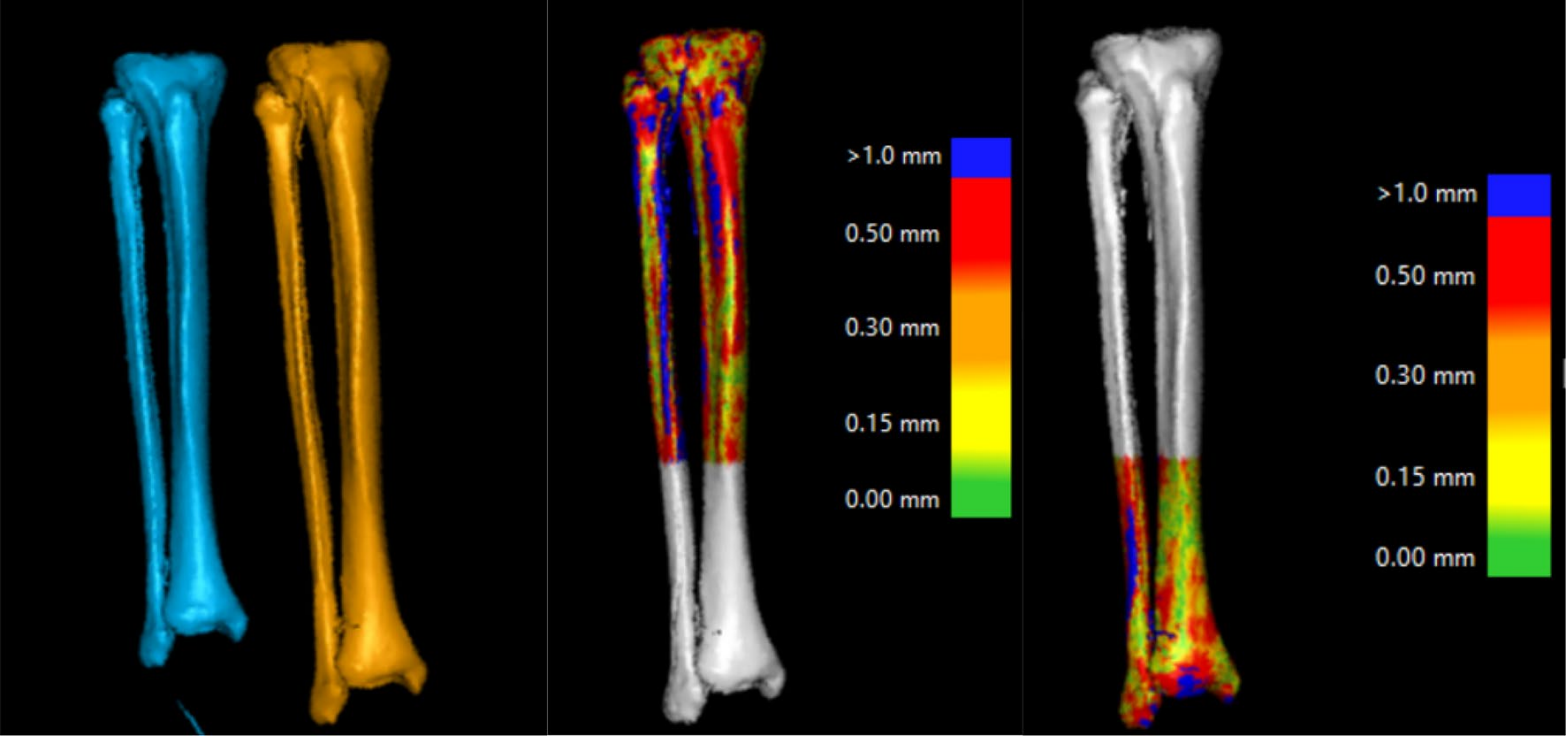
Illustrates merging of mirrored images of the left lower leg and the right lower leg. Initially the proximal parts are merged and assumes as static. Further, the distal parts are merged and assumed as moving.

Two parameters were used to determine optimal segmentation. First, the user indicates the anatomical bony part to be segmented, for example tibia, fibula or distal femur. The Hounsfield unit (HU) values are then used in combination with these clicks to find where one bone ends, and another starts (i.e. optimal segmentation). Further, we used CTMA, a software with high precision to find the relative movement of an object between two different CT-stacks. At first, 100,000 measurement points are spread over the surface of interest. The software then rotates and translates the object in the second CT-stack to match that in the first CT-stack as closely as possible to reach a maximum of surface fusion (Fig. 2).

This is achieved by minimizing the distance between the two groups of points. As the surfaces used to generate fusion are much larger than any artifact areas, the artifacts have limited impact on the matching process. The initial phase is done first for a reference object to create a stationary surface landmark. Afterwards, the movement of the object of interest is measured in the same way. This technique has been previously described by Bakhshayesh et al.^6^.

Following our previous experience using this CTMA, 10,000 points with a mean distance difference between meshes of 0.5 mm or less were chosen.

The proximal part of the right lower legs including the tibia plateau, the fibular head and the proximal diaphyseal areas of tibia and fibula of each STL created 3D volume were merged with the mirrored contralateral side (Fig. 2). These merged images were saved as static, or nonmoving parts, and were used as reference volumes. Furthermore, the distal part of the lower legs including tibia plafond, the distal fibula and the distal diaphyseal part of tibia and fibula were merged and used as moving object.

**Figure 3:**
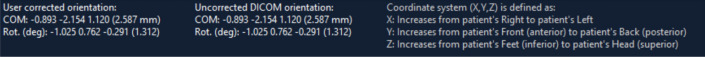
Illustrates automatic calculation of translation of Centre of Mass (COM) and Rotational differences around X, Y and Z axes.

The CTMA gives possibility to measure translational and rotational changes in three different Euler axes (X, Y and Z). According to standards of DICOM movements were defined as per: axis X from left to the right, axis Y from front to back and axis Z from feet to head. Clockwise rotation was assumed positive when looking along the positive axis direction. Changes in translation can be reported either for any special point or for the entire volume of an object based on Centre of Mass (COM). Up to 500,000 points at the moment are placed around the entire object to create a secondary geometric object from which COM is derived. Based on our previous experiences we found using 10 000 points suitable. This COM is quite similar but not absolutely identical to the mathematical center of the object. The reason is because that while the created geometrical pattern of the points is highly close to the object it is never 100% identical. Rotation was reported for the entire geometrical volume (Fig. 3).

### Statistics

As per Root Mean Square Error (RMSE), accuracy was analyzed with mean, median and 95% confidence interval (CI) of the mean^18^. Shapiro–Wilk and Kolmogorov–Smirnov tests were used to test the distribution of normality. Statistical analysis was performed using IBM SPSS Statistics version 25 for Windows. A p-value < 0.05 was considered statistically significant.

## Results

The mean age of patients in our study was 54 ± 20 years. In our material we had six male patients and four females. Of these eight were White British, there was one Black African and the other one from the Middle East. Please see differences in rotation and translation with error bars for translational measurement differences in X, Y and Z-axes of COMs, which are presented in Table 1**.**

**Table 1:** Illustrates translation of COM (Centre of Mass) alongside X, Y and Z axes. Further illustrates rotational changes for all 10 patients, between proximal and distal parts of the merged 3D images of the right lower legs and mirrored images of the left lower legs. SEM; Standard error of the mean.

Patient	COMX (mm) Median: − 1.093 Mean: − 1.626 CI − 2.959–.292 SEM: .589	COMY (mm) Median: − .049 Mean: − .739 CI − 2.974–1.497 SEM: .988	COMZ (mm) Median: .867 Mean: 0.910 CI − 1.418–3.239 SEM: 1.029	ROTX (degrees) Median: − .350 Mean: − .264 CI − .928–.400 SEM: .293	ROTY (degrees) Median: 0.717 Mean: .703 CI .370–1.035 SEM: .147	ROTZ (degrees) Median: − .626 Mean: − 1.184 − 3.444–1.075 SEM: .999
1	0.877	2.944	0.913	1.074	0.94	− 0.526
2	− 5.476	− 4.561	− 0.614	− 1.031	1.43	− 4.293
3	− 0.668	− 0.074	1.373	− 0.313	− 0.086	− 3.019
4	− 2.632	0.779	0.822	0.427	1.184	− 0.5
5	− 1.575	0.093	− 5.403	− 0.105	0.997	− 7.425
6	− − 0.853	− 2.179	0.769	− 1.008	0.536	− 0.725
7	− 3.77	− 6.513	7.955	− 1.829	0.897	1.98
8	− 1.332	− 0.023	1.693	− 0.387	0.487	3.431
9	− 0.301	3.699	− 0.353	1.067	0.23	1.047
10	− 0.527	− 1.553	1.949	− 0.537	0.413	− 1.814

We observed a greater variation in translation of COM in X axis (CI − 2.959–0.292) which was even presented in rotational difference alongside Y axis (CI 0.370–1.035). In other word the right lower legs in our study were more likely to be in varus compared to the left side. We found no statistically significant differences in external/internal rotation or in recurvatum/antecurvatum. All variables were normally distributed. Tests of normality of distribution of the variables are presented in Table 2. The CI of all measurements apart from COM X and ROT Y, crossed zero (Table 1, Fig. 3).

**Table 2:** Illustrates normal distribution of the variables. COM; Centre of Mass. ROT; Rotational differences.

Tests of Normality						
	Kolmogorov-Smirnov^a^			Shapiro–Wilk		
	Statistic	df	Sig	Statistic	df	Sig
COMX	.211	10	.200*	.922	10	.371
COMY	.184	10	.200*	.956	10	.738
COMZ	.275	10	.031	.854	10	.065
ROTX	.132	10	.200*	.954	10	.721
ROTY	.162	10	.200*	.978	10	.950
ROTZ	.158	10	.200*	.970	10	.892

## Discussion

The main finding of our study was that it is possible to use our proposed technique in clinical practice as the software is commercially available and low dose CT scan of the lower extremities is possible^6–8^. The second finding of our study was that, the left and right lower limbs of healthy adults were highly symmetrical in terms of length and rotation. They were further symmetrical in terms of recurvatum and antecurvatum. However, we observed that right lower legs were more likely in varus a fact which has not been mentioned in the literature previously.

Conventional X-ray is widely used to plan lower extremity surgery. The rotational deformity after IM nailing of the lower extremities is not an uncommon issue with a reported incidence of rotational deformity > 10° in up to 20%^1^. The most common technique mentioned in the literature is a method initially introduced by Jakob et al. which was further studied by Jend et al. ^3,19^. This technique is using CT scan over the lower extremities and describes manual measurement techniques which has been criticized for lack of reproducibility which is inherent in all measurement techniques using manual calculation^4^.

In this study we used a novel technique with computerized calculation which minimizes risks of inter- and intra-observer differences and reduces risk of bias.

As this technique is using surface anatomy of the lower legs a high-resolution CT-scan is not necessary and the radiation dosage can be reduced to levels comparable to conventional X-ray^8,20^.

Using our proposed technique with 10 cases we were able to show that there are no rotational (torsional) differences between right and left human lower legs.

However, we found that the right lower leg was more likely in varus. This fact to our knowledge has not previously been mentioned in the literature. Unfortunately, we have no explanation for this finding while we can speculate that future studies would tell us more about this potential difference.

A limitation of our study is it´s limited sample size (10 cases). However, as our data was normally distributed the calculation of mean was not difficult. Of course, if we had larger sample size then the standard error of our means should have been smaller resulting in narrower confidence band.

Another limitation of our proposed technique is that in its current format, it is difficult to use it as an intra-operative tool. This technique in this stage can either be seen as a pre-operative planning tool or for a post-operative control tool.

Unfortunately, the process of importing data from hospital´s PACS and downloading them to a separate server is at the moment a highly time-consuming procedure^7^. This is the explanation of our small sample size.

Ideally with low dose CT scan protocols in place in radiological departments of the hospitals, a low dose CT scan of the lower legs, mirroring 3D images of the unaffected side and fusion of the proximal and distal part of the lower leg will reveal the torsional deformity.

Another benefit of this technique could be for 3D pre-operative templating as we have been able to show that human lower legs are symmetrical.

## Conclusion

A new technique for measurement of the rotational deformities of the lower legs has been proposed. Using this novel image fusion technique, we found that human lower legs are symmetrical. It is therefore appropriate to use our proposed technique in clinical practice to get an accurate and precise answer to whether there are any rotational deformities in the lower legs following our surgical procedures. It is further appropriate to perform pre-operative templating using the unaffected side in for example corrective rotational deformities of the lower legs, fracture surgery etc. Future studies with larger sample size are needed to investigate the finding that the right lower legs were more likely in varus in our study.
